# Sustained Mild Inflammation in Cancer Survivors: Where to from Here?

**DOI:** 10.1093/jncics/pkac054

**Published:** 2022-07-28

**Authors:** Adam K Walker, Raymond J Chan, Janette L Vardy

**Affiliations:** Neuroscience Research Australia (NeuRA), Sydney, Australia; Discipline of Psychiatry and Mental Health, University of New South Wales, Sydney, Australia; Caring Futures Institute, College of Nursing and Health Sciences, Flinders University, Adelaide, Australia; Faculty of Medicine and Health, University of Sydney, Sydney, Australia; Concord Cancer Centre, Concord Repatriation General Hospital, Sydney, Australia

Inflammation is a well-known consequence of many traditional cancer treatments that can serve to enhance antitumor immunity and promote unwanted side effects. These side effects are often sustained long after cancer treatment has ended and may coincide with sustained chronic low-grade systemic inflammation that compromises the health of organs in the body and central nervous system (CNS). Assessment of circulating cytokines and their downstream products are considered to serve as potential biomarkers for systemic inflammation, identifying cancer survivors at risk of inflammation-associated disorders. In relation to the CNS, inflammatory markers are associated with cancer-related fatigue (1); anxiety and depression (2); and, in some studies in women with breast cancer, with cancer-related cognitive impairment (3), but the markers are not seen in men and women with colorectal cancer (4). Despite our increasing understanding of the association between inflammation and these brain-related side effects, we know far less about the effects of specific cancer treatments on inflammatory markers.

We congratulate Bower et al. (5) for conducting the RISE study, with the most recent analysis aiming to fill knowledge gaps by comparing the trajectory of change of circulating inflammatory markers between breast cancer patients receiving standard anticancer therapies. The data are part of a larger longitudinal study investigating cancer-related fatigue in women with stage 0-IIIA breast cancer assessed prior to starting adjuvant or neoadjuvant treatment, approximately 2 weeks after completion of radiation and/or chemotherapy in those who received it, and 6, 12, and 18 months later. Six pro-inflammatory markers were assessed in plasma (tumor necrosis factor–α, interleukin [IL]–6, IL-8, soluble tumor necrosis factor receptor type II, interferon-γ, and C-reactive protein [CRP]) in 192 (71%) of the RISE participants, with the primary aim of evaluating the impact of radiotherapy and chemotherapy on the inflammatory markers (6). When considered broadly, the findings support the contention that inflammation can be sustained above baseline levels for months to years following cancer treatment despite tending to decline posttreatment. Although comparisons by treatment are limited by the sample size, particularly in the chemotherapy-only group (n = 18), the most profound increases occurred in patients receiving chemotherapy or a combination of chemotherapy and radiation. In contrast, no statistically significant changes from baseline were observed for patients who received no radiotherapy or chemotherapy or who had radiotherapy alone.

The longitudinal nature of the study (5) is a major strength, with baseline samples collected prior to (neo)adjuvant treatment. It would have been preferable to exclude participants with stage 0 from the study (or report their results separately), as inflammation may be different in this group, because they do not have invasive cancer, and many do not receive treatment beyond surgery. It is not possible to determine the number of participants with stage 0 vs stage I, as the 2 groups are combined. Additionally, it would have been interesting to have assessed blood samples prior to surgery (other than in the neoadjuvant group [n = 19]), as surgery also induces inflammation that may be variable depending on the invasiveness of the procedure and may contribute to the variability in baseline levels of almost all inflammatory markers between groups. As acknowledged by the authors, the addition of a noncancer control group with inflammatory markers taken at the same time intervals would have further aided interpretation of the findings.

Bower et al.’s (5) findings suggest that chronic low-grade inflammation, as opposed to robust acute inflammatory responses to treatment events, may be responsible for inflammation-associated diseases in survivors after treatment. For instance, with the notable exceptions of IL-8 and CRP, cytokine responses tended to peak in immediate response to treatment, after which they largely resolved without ever quite returning to baseline. Thus, cytokine and CRP concentrations remained mildly elevated but within the normal range. For instance, all samples have CRP values less than 3 mg/L, the typical clinical cutoff range for inflammation possibly because levels of IL-6, CRP’s inducer, stayed as low as the median values of controls in a 2016 meta-analysis of IL-6 levels in cancer patients (7). However, these low levels of CRP are in contrast with a previous study showing mean CRP levels in 734 breast cancer survivors to be much higher (and above the clinical cutoff) 31 months after diagnosis, which along with serum amyloid A, was associated with poorer overall survival and disease-free survival (8). Again, the addition of a control group could help determine whether, although inflammatory markers remain relatively low, they are still elevated compared with age-matched individuals without a cancer history.

Although CRP and cytokine levels in Bower et al.’s (5) study are lower than expected, the persistent low-grade canonical cytokine elevations reported are consistent with findings in noncancer populations that have demonstrated associations between moderate cytokine levels and disorders of the CNS with cognitive, depressive, and anxiety symptoms. This suggests we may gain mechanistic insights from research conducted in other disease types. The difference is that we can infer causation from the singular or combined treatment events experienced by cancer patients. The majority of cytokines that Bower et al. measured are induced predominantly or partially via the nuclear factor kappa B (NF-_Κ_B) transcription pathway. Studies in patients with schizophrenia who, apart from psychosis, share several anxiety, depression, and cognitive symptoms with cancer patients, have identified a subgroup of patients that exhibit elevated brain and blood cell NF-_Κ_B–mediated cytokines. These low-grade cytokine elevations are explained by dysregulation of the NF-_Κ_B, including reduced expression of NF-_Κ_B pathway inhibitors (9). Although speculative, the similarities with cancer survivors in circulating inflammatory biophenotypes and related cognitive symptoms suggest that we may benefit from evaluating the following in cancer survivors: 1) characterization of potential differences in the molecular pathways that regulate inflammation, 2) investigation into the role of anti-inflammatory cytokines and inhibitory mediators of inflammation, and 3) investigation of the response of circulating blood cells to inflammatory stimuli. Bower et al.’s (5) compelling findings direct us toward an exciting opportunity to look beyond inflammatory biomarker assessment and toward function, response, and inhibition—and encourage us to seek new perspectives and creative solutions from other research fields.

In addition to providing a platform for new approaches and discoveries regarding the measurement of inflammatory biomarkers in cancer patients, Bower et al.’s (5) work clarifies the need for a greater understanding of the role and nature of inflammation in other cancer populations. Like this study, most studies evaluating longitudinal changes in inflammation have been in white women with early-stage breast cancer. It is timely to investigate this in more diverse demographic groups, including men and women with other tumor types, different stages of disease, and various anticancer treatments. Our own longitudinal study evaluating inflammatory markers in colorectal cancer survivors found high cytokine levels prior to any chemotherapy (greatest in those with higher stage disease) and persistently elevated cytokine levels at 12 months, compared with healthy controls (4). At a median follow-up of 91 months, there was no evidence that baseline cytokines, or differential ratios of blood components (including neutrophil-to-lymphocyte ratio), taken prior to any chemotherapy and/or radiotherapy, were associated with disease-free survival or overall survival (10). Long-term follow-up 6-12 years after diagnosis in a small subgroup found no difference in CRP levels between healthy controls and the cancer survivors (11). These findings highlight that degree of inflammation and risk of inflammation-associated disease is likely to differ between tumor types and treatments.

Another direction for investigation is related to the evolving treatment landscape. For example, an increasing number of women are receiving T-DM-1. Given there are more prevalent and severe toxicities associated with T-DM1 compared with Trastuzumab (12), future research could explore the relationships between receipts of T-DM1 and inflammatory markers.

As the RISE study focuses on fatigue and longitudinal design (5), we await with interest the results regarding any correlation between inflammatory markers and fatigue trajectory.

## Funding

No funding was used for the writing of this editorial.

## Notes

**Role of the funder:** Not applicable.

**Disclosures:** The authors have no disclosures to declare.

**Author contributions:** AKW, RJC, JLV: writing—original draft, writing—review & editing.

## Data Availability

No new data were generated or analyzed for this editorial.
